# Visuomotor integration deficits are common to familial and sporadic preclinical Alzheimer’s disease

**DOI:** 10.1093/braincomms/fcab003

**Published:** 2021-01-25

**Authors:** Kirsty Lu, Jennifer M Nicholas, Philip S J Weston, Julie C Stout, Alison M O’Regan, Sarah-Naomi James, Sarah M Buchanan, Christopher A Lane, Thomas D Parker, Sarah E Keuss, Ashvini Keshavan, Heidi Murray-Smith, David M Cash, Carole H Sudre, Ian B Malone, William Coath, Andrew Wong, Marcus Richards, Susie M D Henley, Nick C Fox, Jonathan M Schott, Sebastian J Crutch

**Affiliations:** 1 Dementia Research Centre, UCL Queen Square Institute of Neurology, University College London, London, WC1N 3BG, UK; 2 Department of Medical Statistics, London School of Hygiene and Tropical Medicine, London, WC1E 7HT, UK; 3 Turner Institute for Brain and Mental Health, School of Psychological Sciences, Monash University, Melbourne, Victoria, Australia; 4 MRC Unit for Lifelong Health and Ageing at UCL, London, WC1E 7HB, UK; 5 UK Dementia Research Institute at University College London, London, UK; 6 School of Biomedical Engineering and Imaging Sciences, King’s College London, London, SE1 7EU, UK; 7 Department of Medical Physics, University College London, London, WC1E 7JE, UK

**Keywords:** Alzheimer’s disease, amyloid imaging, attention, biomarkers, neurodegeneration, proprioception

## Abstract

We investigated whether subtle visuomotor deficits were detectable in familial and sporadic preclinical Alzheimer’s disease. A circle-tracing task—with direct and indirect visual feedback, and dual-task subtraction—was completed by 31 individuals at 50% risk of familial Alzheimer’s disease (19 presymptomatic mutation carriers; 12 non-carriers) and 390 cognitively normal older adults (members of the British 1946 Birth Cohort, all born during the same week; age range at assessment = 69–71 years), who also underwent β-amyloid-PET/MRI to derive amyloid status (positive/negative), whole-brain volume and white matter hyperintensity volume. We compared preclinical Alzheimer’s groups against controls cross-sectionally (mutation carriers versus non-carriers; amyloid-positive versus amyloid-negative) on speed and accuracy of circle-tracing and subtraction. Mutation carriers (mean 7 years before expected onset) and amyloid-positive older adults traced disproportionately less accurately than controls when visual feedback was indirect, and were slower at dual-task subtraction. In the older adults, the same pattern of associations was found when considering amyloid burden as a continuous variable (Standardized Uptake Value Ratio). The effect of amyloid was independent of white matter hyperintensity and brain volumes, which themselves were associated with different aspects of performance: greater white matter hyperintensity volume was also associated with disproportionately poorer tracing accuracy when visual feedback was indirect, whereas larger brain volume was associated with faster tracing and faster subtraction. Mutation carriers also showed evidence of poorer tracing accuracy when visual feedback was direct. This study provides the first evidence of visuomotor integration deficits common to familial and sporadic preclinical Alzheimer’s disease, which may precede the onset of clinical symptoms by several years.

## Teaser

Using a computerized circle-tracing task with direct and indirect visual feedback, Lu et al. report the first evidence of visuomotor integration deficits in both familial and sporadic preclinical Alzheimer’s disease groups, suggesting that these deficits might precede the onset of clinical symptoms by several years.

## Graphical Abstract

**Graphical Abstract:**
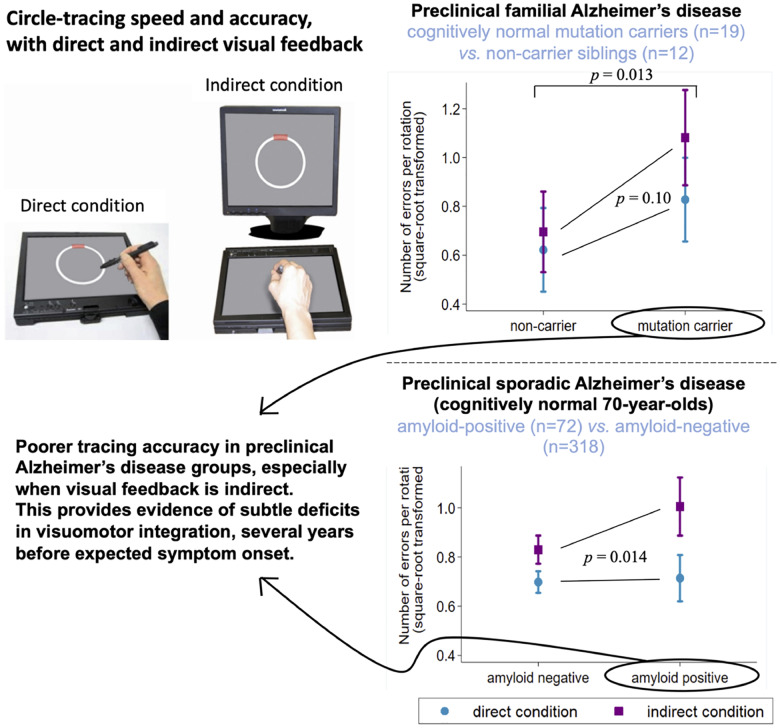

## Introduction

Visuomotor integration describes the ability to combine visual information with motor output. Although visuomotor integration difficulties have received relatively little attention in Alzheimer’s disease—compared to the well-established impairments in memory, orientation and executive functions—deficits have been observed in Alzheimer’s patients and individuals at high Alzheimer’s disease risk, particularly under conditions where visual feedback is indirect (e.g. transformed with respect to the plane of movement) (Tippett and Sergio, 2006; Tippett *et al.*, 2007; Hawkins and Sergio, 2014, 2016; Hawkins *et al.*, 2015; Bartoli *et al.*, 2017; Rogojin *et al.*, 2019). It has been proposed that the early emergence of such deficits in Alzheimer’s disease may be linked to the vulnerability of the posterior parietal cortex—a region understood to be particularly important for co-ordinating information about eye and hand movements (Tippett and Sergio, 2006; Hawkins and Sergio, 2014). Furthermore, visuomotor integration tasks are inherently multi-modal and multi-tasking in nature, posing a higher-order cognitive challenge in common with other neuropsychological tests that have shown sensitivity to early decline, such as the commonly used Digit-Symbol test [e.g. Mormino *et al.* (2017)].

However, there has been little investigation of whether subtle changes in visuomotor integration are detectable during the preclinical stages of Alzheimer’s disease—which may extend two to three decades before symptom onset (Villemagne *et al.*, 2013; McDade *et al.*, 2018)—and how these may relate to accumulation of pathology. It is also unclear whether common visuomotor deficits emerge in the preclinical stages of familial (autosomal dominant) and sporadic Alzheimer’s disease, given the different age profiles of these two populations and ongoing debates about the extent of pathological and phenotypic differences between them [e.g. motor abnormalities such as myoclonus (Joshi *et al.*, 2012; Day *et al.*, 2016; Ryan *et al.*, 2016)].

Using a computerized circle-tracing task that has detected visuomotor integration deficits in presymptomatic Huntington’s disease up to a decade before expected symptom onset (Say *et al*., 2011), we investigated whether similar subtle deficits were present in two preclinical Alzheimer’s groups—presymptomatic familial Alzheimer’s disease (FAD) mutation carriers, and cognitively normal older adults with β-amyloid pathology. We hypothesized that these groups would trace less accurately than controls, particularly when visual feedback was indirect (requiring spatial transformation between planes). We also aimed to investigate associations between task performance and other neuroimaging measures: whole-brain volume and white matter hyperintensity volume [WMHV, a marker of cerebral small vessel disease (Sudre *et al.*, 2015)].

## Materials and methods

Thirty-one asymptomatic individuals (Clinical Dementia Rating = 0) at 50% risk of FAD (due to having an affected parent with a mutation in the *PSEN1* or *APP* gene) were assessed between February 2015 and March 2016 as part of a longitudinal FAD study at University College London (O’Connor *et al.*, 2020). Participants underwent genetic testing to determine mutation status, but the results were available only to statisticians; participants and study staff remained blind to mutation status. As mutation carriers are expected to develop symptoms at around the same age as their affected parent, years to expected onset was calculated by subtracting the age that a participant’s affected parent developed symptoms (based on a semi-structured interview of family members) from the participant’s current age. Thus, a larger negative value indicates greater years before expected onset. No participants had evidence of any movement disorder on neurological examination.

Between May 2015 and January 2018, 502 members of the MRC National Survey of Health and Development (also known as the British 1946 Birth Cohort; all born during the same week in March 1946) were assessed at University College London for the Insight 46 sub-study. Recruitment and assessment protocols have been published previously (Lane *et al.*, 2017; James *et al.*, 2018). In brief, participants underwent clinical examination, neuroimaging (combined MRI and β-amyloid PET) and a multi-domain neuropsychological battery comprising standard paper-and-pencil tests and more novel computerized tasks (Lane *et al.*, 2017; Lu *et al.*, 2019, 2020). This analysis included all cognitively normal participants with complete neuroimaging data and no clinical tremor disorder [*n* = 390, see criteria for normal cognition and reasons for missing neuroimaging data in Lu *et al.* (2019)].

Both studies were approved by London Queen Square Research Ethics Committee. All participants provided written informed consent according to the declaration of Helsinki.

We use the short-hand term ‘preclinical groups’ to refer to FAD mutation carriers and amyloid-positive Insight 46 participants (see definition of amyloid-positivity), but note the non-equivalence of these two groups in terms of prognosis: FAD mutations are almost fully penetrant, whereas amyloid-positivity in older adults indicates increased risk of Alzheimer’s disease but it is not an accurate predictor on an individual basis (Brookmeyer and Abdalla, 2018).

### Circle-tracing task

**Figure 1:**
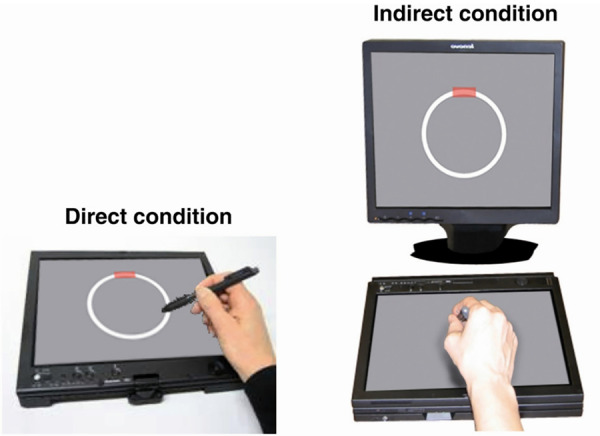
**Circle-tracing apparatus. This figure is reprinted from Say et al. (2011) with permission from Elsevier.** The additional box and cape to cover the participant’s arm in the indirect condition are not shown. The tablet displayed a 90-mm diameter circle and 5-mm thick annulus; the monitor displayed a 143-mm diameter circle and 9-mm thick annulus. The tablet and monitor were positioned to achieve a consistent horizontal diameter of ∼13° of visual angle. The red rectangle indicates the starting point from which participants were instructed to begin tracing.

The circle-tracing task was presented on a Lenovo ThinkPad-X61 tablet with an additional monitor (Fig. 1). Participants were instructed to trace clockwise round the circle using a stylus as quickly and accurately as possible, with their dominant hand. A thin line appeared to show their tracing path. In the direct condition, participants could see their hand and their tracing path on the tablet. In the indirect condition, the tablet was covered by a box with the front open to allow participants to put their hand inside, and participants wore a cape covering the arms, and hence they had no direct visual feedback but could view a copy of the circle and the tracing path on the monitor. Each trial lasted 45 s.

In the FAD study, 12 trials were administered (ordered DDDIIIDDDIII; D=direct, I=indirect). The last six trials included dual-task serial subtraction similar to the previously published studies (Vaportzis *et al.*, 2014, 2015): participants were asked to count backwards in threes (starting from 99, 98, 97, 96, 95 and 94). If they reached zero or near zero, they were instructed to begin again from the starting number. No instructions were given about whether to prioritize the circle-tracing or the serial subtraction task. For Insight 46, a 6-trial version was administered (DIIDDI) with dual-task subtraction during all trials. This shorter version does not permit evaluation of dual-task costs, but it allowed us to administer the most challenging version of circle-tracing within our time constraints. The order of trials was chosen to allow participants to familiarize themselves first with the easier direct condition [as described the in previously published studies (Vaportzis *et al.*, 2014, 2015)].

The following trial-by-trial outcomes were derived:

- Number of rotations (index of tracing speed).
- Number of errors per rotation (index of tracing accuracy). An error was recorded whenever the stylus deviated outside the annulus for >100 ms.
- Subtraction rate (responses per second).
- Number of incorrect subtractions.

### Insight 46 variables

As previously described (Lane *et al.*, 2017, 2019), β-amyloid-PET and multi-modal MRI data were collected during a 60-min scanning session on a single Biograph mMR 3 T-PET/MRI scanner (Siemens Healthcare, Erlangen), with intravenous injection of 370 MBq of 18 F-Florbetapir (Amyvid). β-Amyloid deposition was quantified using the Standardized Uptake Value Ratio (SUVR), with a cut-point for Aβ-positivity at SUVR >0.6104. Whole-brain volume was generated from 3D T1-weighted MRI using automated segmentation with manual editing (Leung *et al.*, 2011). Total intracranial volume was generated using statistical parametric mapping (SPM12; http://www.fil.ion.ucl.ac.uk/spm; accessed 29 January 2021) (Malone *et al.*, 2015). Global WMHV was generated using an automated segmentation algorithm (Sudre *et al.*, 2015) followed by visual quality control, including subcortical grey matter but excluding infra-tentorial regions. *APOE* genotype was categorized as ε4-carrier or ε4-non-carrier.

As previously described (Lu *et al.*, 2019), childhood cognitive ability was measured at 8 years (or 11 or 15 years if earlier data were missing) and highest educational qualification was recorded at 26 years—this was converted to the equivalent years of full-time education, for consistency with the FAD sample. Socioeconomic position was derived from participant’s own occupation at 53 years, coded according to the UK Registrar General's Standard Occupational Classification, and categorized as manual or non-manual.

### Statistical analyses

As participants occasionally did not trace continuously for 45 s as instructed, we excluded trials where the total tracing time was <34.7 s (3 *SD* below the mean): 1.1 and 1.3% of trials from the FAD and Insight 46 data sets, respectively (≤2 trials per participant).

Standard techniques were used to assess data normality and model fit. A log transformation was applied to the number of rotations, and a square-root transformation to the number of errors per rotation, consistent with the previously published studies (Say *et al.*, 2011).

For tracing speed, tracing accuracy and subtraction rate, we fitted multivariable regression models using generalized estimating equations, assuming a normal distribution for the dependent variable and an identity link, with an exchangeable correlation structure and robust standard errors to allow for the correlation between repeated measures of the same participant.

For subtraction accuracy, a generalized estimating equations logistic regression model was used with an independent correlation structure and robust standard errors. The outcome was the number of incorrect responses, treated as a proportion of the total number of responses.

For the FAD data set, model predictors were condition (direct versus indirect), task (dual versus single), age, sex, education and mutation status (carrier versus non-carrier). For Insight 46, model predictors were condition (direct versus indirect), age, sex, childhood cognitive ability, education, socioeconomic position, handedness (left versus right), amyloid status (positive versus negative), WHMV, whole-brain volume and *APOE-*ε4 (ε4-carrier versus ε4-non-carrier). To adjust for the correlation between brain volumes and head size, total intracranial volume was included as a covariate. To investigate specific visuomotor integration deficits (i.e. disproportionately poorer performance in the indirect condition), we tested for interactions between circle-tracing condition and predictors of interest.

For the outcomes where statistically significant differences were observed between preclinical groups and controls (i.e. circle-tracing accuracy and subtraction speed), we conducted additional analyses to investigate whether poorer performance was correlated with continuous measures of greater pathological burden. For FAD mutation carriers, years to expected onset can be used as a proxy for pathological burden; to investigate whether closer proximity to expected onset predicted poorer performance, the models described above were re-run in mutation carriers only with an additional predictor of years to expected onset. In Insight 46 participants, we tested whether greater burden of amyloid pathology was associated with poorer performance by rerunning the models describe above, replacing dichotomized amyloid status with continuous SUVR. We additionally refitted the models using a linear spline with a knot at the cut-point for amyloid-positivity, to explore whether the slope differed for amyloid-positive and amyloid-negative groups. Regression coefficients for SUVR are quoted per 0.1 increment.

As speed–accuracy trade-offs have been noted on motor tasks including circle-tracing (Nagengast *et al.*, 2011; Vaportzis *et al.*, 2014; Peternel *et al.*, 2017), we investigated between-subject speed–accuracy trade-offs (i.e. whether participants who traced more quickly tended to trace less accurately, and whether participants who subtracted more quickly tended to make more subtraction errors). We calculated each participant’s mean number of rotations, mean number of errors per rotation, mean subtraction rate and mean subtraction error rate across all available trials, then used Pearson’s correlation (for circle-tracing) and Spearman’s correlation (for subtraction—due to highly skewed error rate) to examine the relationships between the speed and the accuracy measures.

Supplementary Material describes about the investigation of ‘dual-task cost’ in FAD participants and comparison of performance between FAD and Insight 46 samples.

Analyses were conducted using Stata-15 (StataCorp). Statistical significance was set at *P*** **<** **0.05 for associations and *P*** **<** **0.1 for interactions.

### Data availability

Insight 46 data are available via https://skylark.ucl.ac.uk/NSHD/doku.php; accessed 29 January 2021. FAD data are available on reasonable request.

## Results

Participant characteristics and descriptive statistics for outcome measures are listed in Table 1. For details of performance on standard neuropsychological tests, see Supplementary Material (FAD) and Lu *et al.* (2019) (Insight 46).

**Table 1:** Participants’ characteristics and descriptive statistics for the circle-tracing task

	**FAD participants** ^a^		**Insight 46 participants** ^a^	
	Mutation carriers	Non-carriers	β-Amyloid positive	β-Amyloid negative
*N*	19	12	72	318
Sex: % female	47.4	50.0	45.8	49.7
Age (years)^b^: mean (*SD)*	39.0 (5.2)	39.7 (8.2)	70.6 (0.66)	70.6 (0.70)
Handedness: % right	100	100	88.9	90.9
Years to expected onset: mean (*SD)*	−7.0 (4.5)			
Years of education: mean (*SD)*	13.6 (2.6)	15.0 (2.4)	13.3 (2.1)	13.6 (2.1)
Childhood cognitive ability (*Z*-score)^c^: mean (*SD)*			0.43 (0.75)	0.41 (0.74)
Socioeconomic position: % manual			13.9	14.2
SUVR: median, *IQR*, (range)			0.68, *0.64–0.72*, (0.61–0.87)	0.53, *0.51–0.56*, (0.47–0.61)
WMHV (cm^3^): median, *IQR*, (range)			3.3, *1.8–6.9*, (0.3–33.7)	2.7, *1.5–6.1*, (0.3–32.8)
Whole-brain volume (cm^3^): mean, *SD*, (range)			1119, *104*, (819–1326)	1099, *98*, (860–1494)
*APOE* genotype: % ε4-carrier			61.1	23.6
TASK OUTCOMES^d^				
Number of rotations: median (IQR)				
Dual-task	7.8 (5.2–10.3)	6.4 (4.5–8.0)	4.4 (3.2–6.5)	4.4 (2.9–6.4)
Single-task	10.4 (6.3–12.8)	7.0 (5.7–10.0)		
Errors per rotation: median (IQR)				
Dual-task	0.95 (0.49–1.54)	0.32 (0.18–0.52)	0.94 (0.51–1.40)	0.73 (0.37–1.30)
Single-task	1.35 (1.09–1.95)	0.46 (0.33–0.88)		
Subtraction rate (responses per second): median (IQR)	0.43 (0.24–0.58)	0.65 (0.46–0.75)	0.47 (0.39–0.55)	0.50 (0.37–0.63)
Subtraction error rate (%): median (IQR)	1.7 (0.5–5.4)	0.9 (0–1.8)	1.0 (0–3.5)	1.1 (0–2.9)

FAD, familial Alzheimer’s disease; IQR, interquartile range; SD, standard deviation; WMHV, white matter hyperintensity volume.

a *χ* ^2^, two-tailed *t*-tests, and rank-sum tests were used to test for differences between the groups (mutation carriers versus non-carriers; amyloid-positive versus amyloid-negative) for the demographic, biomarker and clinical variables: the only variable with a statistically significant difference was *APOE*-ε4 between amyloid-positive and amyloid-negative (*P* < 0.0001).

b For Insight 46, age was calculated based on the date that the cognitive assessment was carried out [while assessments were typically completed on one day, 62 participants had to have their scans rescheduled for a later date, with a median interval of 49 days (range, 1–216 days)].

c *Z*-scores for childhood cognitive ability were based on the full National Survey of Health and Development (NSHD) cohort of *N* = 5362, and hence the mean for Insight 46 participants indicates that they had higher childhood cognitive ability on average than their peers not recruited to this sub-study.

d Although statistical analyses were carried out on the trial-by-trial data, each participant’s mean score across trials was calculated for each outcome for the purposes of this table (combined across the direct and indirect visual feedback conditions), to illustrate the distributions of performance.

**Figure 2:**
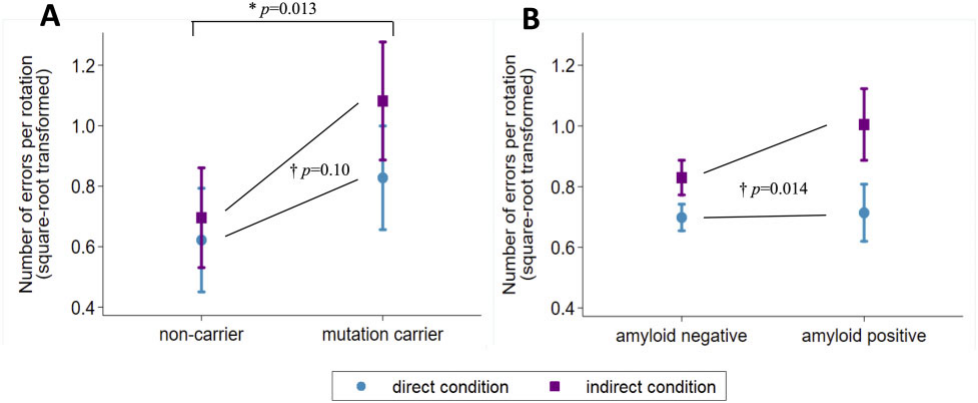
**Circle-tracing errors in the conditions of direct and indirect visual feedback for (**A**) FAD mutation carriers versus non-carriers, (**B**) amyloid-positive versus amyloid-negative older adults.** Plots show marginal means and 95% confidence intervals from the multivariable regression models. *Mutation carriers made more errors than non-carriers overall (*P*-value is for the main effect of mutation status from the regression model reported in Table 2). †The increase in errors in the indirect condition (compared to the direct condition) was greater in the preclinical Alzheimer’s groups (FAD mutation carriers; amyloid-positive) than controls (non-carriers; amyloid-negative) (*P*-values are for interaction tests between group and condition, from the multivariable regression models). This figure may be compared with Fig. 2B in Say et al. (2011), which shows a similar pattern in presymptomatic Huntington’s disease: the equivalent square-root-transformed values for presymptomatic Huntington’s mutation carriers (on average 8.7 years from expected onset) were ∼0.5 versus ∼1.2 (direct versus indirect), compared to controls with ∼0.5 versus ∼0.9 (direct versus indirect). In (**B**), the plotted means and the *P*-values are identical to two decimal places if the model does not include adjustment for WMHV, whole-brain volume and *APOE*-ε4. See Supplementary Fig. 1 for a version of this graph that includes individual-level data.

As expected, tracing was slower and less accurate in the indirect condition than the direct condition (Table 2 and Fig. 2). Despite tracing at a similar speed, FAD mutation carriers traced less accurately than non-carriers; this difference was exaggerated in the indirect condition, although this interaction was not statistically significant (interaction coefficient 0.18 [95% CIs: −0.04, 0.40] *P*** **=** **0.10) (Tables 1 and 2, Fig. 2A and see also Supplementary Fig. 1A). In Insight 46 participants, there was no overall association between amyloid status and tracing speed or accuracy (Table 2), but there was an interaction by which amyloid-positive participants traced disproportionately less accurately (i.e. made more errors) in the indirect condition (interaction coefficient 0.16 [95% CIs: 0.03, 0.29] *P*** **=** **0.014) (Fig. 2B and see also Supplementary Fig. 1B). Furthermore, both preclinical groups were slower at subtraction, compared to control groups, but did not significantly differ in subtraction accuracy (Tables 1 and 2).

**Table 2:** Predictors of performance on circle-tracing and serial subtraction task in FAD and Insight 46 participants

Predictor	FAD participants (*n* = 31)				Insight 46 participants (*n* = 390)			
	**Number of rotations** ^a^	**Errors per rotation** ^b^	**Subtraction rate** ^c^	**Subtraction errors** ^d^	**Number of rotations** ^a^	**Errors per rotation** ^b^	**Subtraction rate** ^c^	**Subtraction errors** ^d^
	coefficient (95% CIs)	coefficient (95% CIs)	coefficient (95% CIs)	*OR* (95% CIs)	coefficient (95% CIs)	coefficient (95% CIs)	coefficient (95% CIs)	*OR* (95% CIs)
Condition (direct as reference)	**0.49** ^**^ (0.44, 0.54) *P* < 0.0001	**0.18** ^**^ (0.06, 0.31) *P* = 0.004	0.02 (−0.00, 0.04) *P* = 0.084	0.43 (0.18, 1.04) *P* = 0.060	**0.55** ^**^ (0.54, 0.56) *P* < 0.0001	**0.16** ^**^ (0.11, 0.21) *P* < 0.0001	**−0.04** ^**^ (−0.04, −0.03) *P* < 0.0001	1.09 (0.94, 1.25) *P* = 0.25
Task (single as reference)	**0.83** ^**^ (0.77, 0.90) *P* < 0.0001	**−0.17** ^**^ (−0.24, −0.10) *P* < 0.0001						
Sex (female as reference)	1.15 (0.79, 1.67) *P* = 0.46	0.04 (−0.17, 0.26) *P* = 0.70	−0.03 (−0.18, 0.12) *P* = 0.67	1.13 (0.44, 2.91) *P* = 0.81	1.01 (0.87, 1.18) *P* = 0.90	−0.06 (−0.15, 0.04) *P* = 0.27	**0.09** ^**^ (0.05, 0.13) *P* < 0.0001	1.18 (0.79, 1.76) *P* = 0.42
Age (per year)	0.98 (0.96, 1.01) *P* = 0.22	−0.01 (−0.03, 0.01) *P* = 0.38	0.00 (−0.01, 0.01) *P* = 0.95	0.97 (0.91, 1.04) *P* = 0.38	**1.15** ^**^ (1.05, 1.27) *P* = 0.004	0.05 (−0.00, 0.11) *P* = 0.074	0.02 (−0.01, 0.04) *P* = 0.15	1.06 (0.83, 1.36) *P* = 0.63
Education (per year)	**0.92** ^*^ (0.86, 0.98) *P* = 0.014	−0.04 (−0.08, 0.00) *P* = 0.051	0.00 (−0.02, 0.03) *P* = 0.64	1.10 (0.90, 1.35) *P* = 0.33	0.97 (0.94, 1.01) *P* = 0.14	−0.02 (−0.04, 0.01) *P* = 0.14	0.00 (−0.01, 0.01) *P* = 0.58	**0.89** ^*^ (0.80, 0.98) *P* = 0.023
Mutation status (non-carrier as reference)	1.02 (0.72, 1.46) *P* = 0.90	**0.30** ^*^ (0.06, 0.53) *P* = 0.013	**−0.18** ^*^ (−0.33, −0.04) *P* = 0.015	1.74 (0.57, 5.36) *P* = 0.33				
Amyloid status^e^ (negative as reference)					1.05 (0.90, 1.22) *P* = 0.55	0.10 (−0.01, 0.20) *P* = 0.066	**−0.06** ^**^ (−0.09, −0.02) *P* = 0.004	1.25 (0.87, 1.79) *P* = 0.22
WMHV (per 10 ml)					0.90 (0.81, 1.00) *P* = 0.055	0.03 (−0.05, 0.10) *P* = 0.46	-0.02 (−0.05, 0.00) *P* = 0.11	1.22 (0.93, 1.60) *P* = 0.15
Whole-brain volume (per 10 ml)					**1.02** ^**^ (1.01, 1.04) *P* = 0.002	−0.00 (−0.01, 0.01) *P* = 0.91	**0.004** ^*^ (0.001, 0.007) *P* = 0.016	1.00 (0.97, 1.03) *P* = 0.87
Childhood cognitive ability (per *Z*-score)					0.97 (0.89, 1.06) *P* = 0.53	−0.06 (−0.12, 0.00) *P* = 0.052	**0.05** ^**^ (0.02, 0.07) *P* < 0.0001	**0.71** ^**^ (0.59, 0.87) *P* = 0.001
Socioeconomic position (manual as reference)					1.12 (0.93, 1.34) *P* = 0.24	−0.03 (−0.15, 0.08) *P* = 0.55	**0.04** ^*^ (0.00, 0.08) *P* = 0.043	1.17 (0.76, 1.78) *P* = 0.48
*APOE*-ε4 (non-carriers as reference)					1.00 (0.87, 1.16) *P* = 0.95	−0.02 (−0.12,0.07) *P* = 0.62	0.01 (−0.02, 0.05) *P* = 0.48	0.80 (0.60, 1.08) *P* = 0.15

* Significant at *P* < 0.05.

** Significant at *P* < 0.01.

Multivariable regression models were used and hence each association is independent of all others. In addition to the predictors listed, the Insight 46 models also included total intracranial volume and handedness. (Handedness was not applicable to the FAD models since all FAD participants were right-handed.)

FAD, familial Alzheimer’s disease; CI, confidence interval; WMHV, white matter hyperintensity volume.

a Number of rotations was log-transformed and coefficients are expressed in exponentiated form, e.g. a coefficient of 1.5 would mean 50% more rotations.

b Number of rotations per rotation was square-root transformed.

c Subtraction rate is in units of responses per second.

d Odds ratios >1 reflect higher error rates.

e The coefficients for amyloid status are essentially unchanged if the model does not adjust for WMHV, whole-brain volume and *APOE*-ε4.

In Insight 46 participants, greater WMHV also predicted disproportionately poorer tracing accuracy in the indirect condition, independently of amyloid (interaction coefficient 0.10 [95% CIs: 0.01, 0.20], *P*** **=** **0.037). In addition, larger brain volume predicted faster tracing and faster subtraction; higher childhood cognitive ability was associated with faster and more accurate subtraction; socioeconomic position and education had independent effects on subtraction rate and accuracy respectively; and males subtracted faster than females (Table 2).

As there is some evidence of sex differences in neural control of complex movements (Gorbet and Sergio, 2007), and a previous study found that only females showed an association between dementia risk (based on family history of Alzheimer’s disease) and visuomotor integration impairments (Rogojin *et al.*, 2019), we conducted a *post hoc* analysis to test whether males and females differed in terms of the interaction between condition and group on circle-tracing accuracy. In the FAD sample, there was a three-way interaction between condition, group and sex (interaction coefficient = −0.42 [95% CIs: −0.82, −0.02], *P*** **=** **0.040), such that female mutation carriers had disproportionately poorer tracing accuracy in the condition of indirect visual feedback (estimated interaction coefficient between condition and mutation status for females = 0.40 [0.12–0.67], *P*** **=** **0.005), but this effect was not seen among males (estimated interaction coefficient between condition and mutation status for males = −0.02 [−0.32 – 0.27], *P*** **=** **0.88). However, in the Insight 46 sample, there was no evidence of such a sex difference (three-way interaction coefficient between condition, sex, and amyloid status = 0.07 [−0.19, 0.32], *P*** **=** **0.60), as males and females had similar estimated interaction coefficients between condition and amyloid status (males: 0.19 [0.02, 0.37], *P*** **=** **0.032; females: 0.12 [−0.06, 0.31], *P*** **=** **0.19).

### Associations with continuous measures of pathological burden

There was also some evidence of associations between continuous measures of greater pathological burden and poorer circle-tracing accuracy. In FAD mutation carriers, tracing accuracy decreased with closer proximity to expected symptom onset, although the effect was not statistically significant, likely due to the small sample size [0.03 increase per year (errors per rotation, square-root transformed) [95% CIs: −0.00, 0.07], *P*** **=** **0.086]. In the Insight 46 sample, results across the full range of SUVR were consistent with the analyses using dichotomized amyloid status: while there was no statistically significant association between SUVR and tracing accuracy overall (regression coefficient = 0.051 [95% CIs: −0.006, 0.107], *P*** **=** **0.079), there was an interaction by which higher SUVR was associated with disproportionately poorer tracing accuracy (i.e. more tracing errors) in the condition of indirect visual feedback (interaction coefficient between SUVR and condition = 0.087 [95% CIs: 0.021, 0.153], *P*** **=** **0.010). Re-running the model in the indirect condition alone confirmed that higher SUVR was associated with greater tracing errors in this condition (regression coefficient = 0.091 [95% CIs: 0.021, 0.161], *P*** **=** **0.010) and a spline analysis suggested that the slope of this association was steeper in amyloid-positive individuals, although the associations were not statistically significant in the amyloid groups separately (amyloid-positive: regression coefficient = 0.127 [95% CIs: −0.016, 0.270], *P*** **=** **0.082; amyloid-negative: regression coefficient = 0.055 [95% CIs: −0.100, 0.210], *P*** **=** **0.488).

Higher SUVR also predicted slower subtraction rate in Insight 46 participants (regression coefficient = −0.025 [95% CIs: −0.045, −0.006], *P*** **=** **0.010), and a spline analysis revealed that the slope of this association differed for amyloid-positive and amyloid-negative participants, with higher SUVR associated with slower subtraction among amyloid-positive participants (regression coefficient = −0.037 [95% CIs: −0.074, −0.000], *P*** **=** **0.047), but not among amyloid-negative (regression coefficient = −0.014 [95% CIs: −0.055, 0.028], *P*** **=** **0.522). However, FAD mutation carriers showed no evidence of an association between years to expected onset and subtraction rate, (−0.010 per year [95% CIs: −0.031, 0.014], *P*** **=** **0.441).

### Speed–accuracy trade-offs

**Figure 3:**
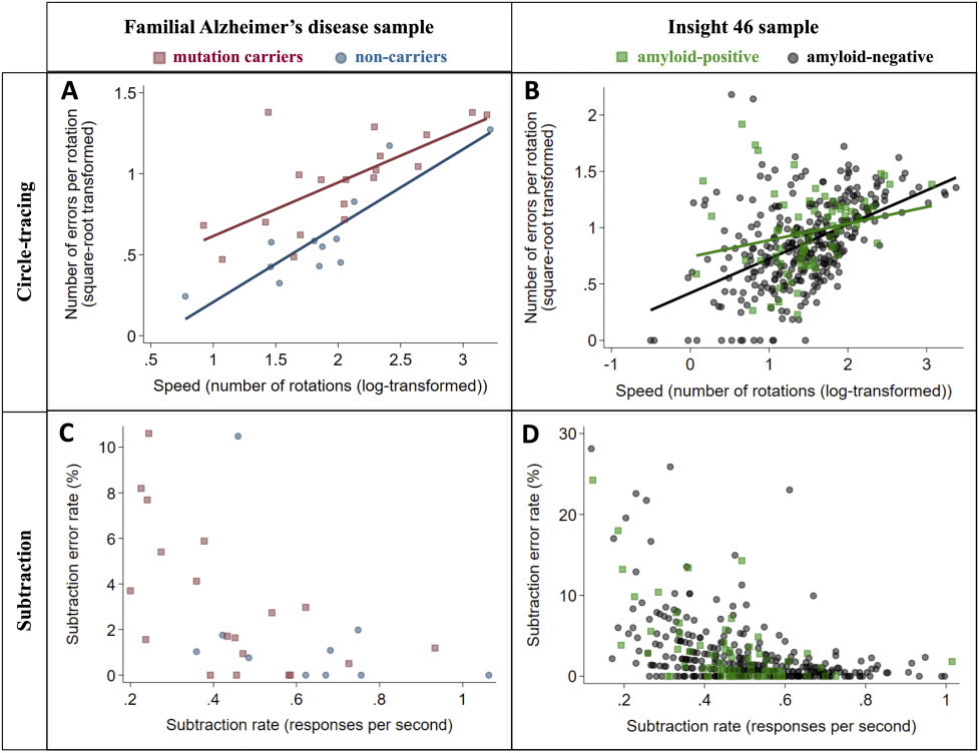
**Relationships between speed and accuracy for circle-tracing and subtraction in the FAD (*n* = 31) and Insight 46 (*n* = 390) samples.** Markers show each participant’s mean across all trials. A speed–accuracy trade-off was observed for circle-tracing, as there was a positive correlation between number of rotations and number of errors per rotation (**A** and **B**). For the subtraction task, faster subtraction correlated with lower error rate (**C** and **D**). Correlation coefficients for each group were as follows: (**A**) (mutation carriers *r* = 0.77, *P* = 0.0001; non-carriers *r* = 0.86, *P* = 0.0004); (**B**) (amyloid-positive *r* = 0.23, *P* = 0.0499; amyloid-negative *r* = 0.49, *P* < 0.0001); (**C**) (mutation carriers *ρ* = −0.63, *P* = 0.0035; non-carriers *ρ* = −0.43, *P* = 0.21); (D) (amyloid-positive *ρ* = −0.50, *P* < 0.0001; amyloid-negative *ρ* = −0.49, *P* < 0.0001).

Speed-accuracy trade-offs were a notable feature of circle-tracing performance in both the FAD and the Insight 46 samples, with a positive correlation between number of rotations and number of errors per rotation (FAD: *r* = 0.75, *P*** **<** **0.0001; Insight 46: *r* = 0.45, *P*** **<** **0.0001) (Fig. 3). This indicates that participants tended to prioritize accuracy at the expense of speed, or vice versa, consistent with the previously published studies (Nagengast *et al.*, 2011; Vaportzis *et al.*, 2014; Peternel *et al.*, 2017). This may account for the counter-intuitive association between older age and faster tracing in the Insight 46 sample (Table 2), as there was a trend for older participants to make more tracing errors (*P*** **=** **0.074, Table 2), suggesting that they may have adopted a less cautious approach. Similarly, the association between higher education and slower tracing in the FAD sample (Table 2) should be interpreted in the context of the association between higher education and fewer tracing errors (*P*** **=** **0.051, Table 2).

In contrast, no speed–accuracy trade-off was observed for subtraction; in fact, there was a negative correlation between subtraction rate and subtraction error rate in both samples (FAD: *ρ* = −0.59, *P*** **=** **0.0008; Insight 46: *ρ* = −0.48, *P*** **<** **0.0001) (Fig. 3). This indicates that participants who found subtraction relatively difficult tended to be both slower and more error-prone.

## Discussion

To our knowledge, this is the first study to report an association between β-amyloid pathology and visuomotor integration deficits, and also the first to show that such deficits may be common to familial and sporadic preclinical Alzheimer’s disease. As hypothesized, both preclinical groups (FAD mutation carriers; amyloid-positive older adults) showed evidence of subtly impaired visuomotor integration, indicated by disproportionately poorer circle-tracing accuracy when visual feedback was indirect, although results for the familial group should be interpreted with caution due to low statistical power. This mirrors the result in presymptomatic Huntington’s mutation carriers (Say *et al.*, 2011). Although speed–accuracy trade-offs were observed as expected on the circle-tracing task, this does not explain the poorer accuracy of the preclinical groups, since we did not find evidence of group differences in tracing speed.

Despite the different age profiles of the two groups, individuals within them who will go on to develop Alzheimer’s disease may be at similar points along the preclinical continuum: FAD mutation carriers were on average 7 years before expected symptom onset, and Insight 46 participants (age, ∼70 years) were around a decade away from the average age of onset of sporadic Alzheimer’s disease [76 years in *APOE*-ε4 homozygotes, 84 years in ε4-non-carriers (Liu *et al.*, 2013)]. Regarding whether these early deficits in visuomotor integration may precede other cognitive changes, mutation carriers performed normally on most standard tests—the only exceptions being, interestingly, tasks with similar spatial transformation cognitive demands to circle-tracing (Supplementary Material). Their predominantly normal cognitive performance and lack of observed memory deficits are consistent with a longitudinal analysis from the Dominantly Inherited Alzheimer Network which found that decline on a cognitive composite did not emerge until ∼3 years before expected symptom onset (McDade *et al.*, 2018). Also, the majority of mutation carriers in our sample carried *PSEN1* mutations, which are associated with less medial temporal lobe atrophy and more cortical loss than *APP* mutations or sporadic Alzheimer’s disease (Pilotto *et al.*, 2013; Scahill *et al.*, 2013). In contrast, amyloid-positive Insight 46 participants showed subtle deficits in memory, non-verbal reasoning, and consistency of reaction time (Lu *et al.*, 2019, 2020). Their visuomotor integration deficit (i.e. the mean difference between amyloid-positive and amyloid-negative groups in circle-tracing accuracy with indirect visual feedback—equivalent to 0.35 SD) was of a similar magnitude to their deficits in non-verbal reasoning (0.39 SD) and reaction time consistency (0.37 SD), but greater than on the Preclinical Alzheimer Cognitive Composite (0.17 SD) (Lu *et al.*, 2019, 2020).

A notable difference between the two samples was that FAD mutation carriers traced less accurately than non-carriers across the whole task, whereas for amyloid-positive older adults their deficit was restricted to the condition of indirect feedback. This could reflect the higher reported prevalence of neurological and movement abnormalities (e.g. myoclonus and pyramidal signs) in familial compared to sporadic Alzheimer’s disease, although debates continue over the extent of such phenotypic differences (Joshi *et al.*, 2012; Tang *et al.*, 2016; Vöglein *et al.*, 2019).

While this was a cross-sectional study, our results imply that this task may have potential for tracking decline, as greater burden of amyloid pathology (continuous measure) correlated with greater visuomotor integration difficulties and slower subtraction, with evidence that these associations may be stronger in amyloid-positive participants. Similarly, visuomotor integration in FAD mutation carriers appeared to be worse with closer proximity to expected onset, although this was inconclusive due to the small sample size. Another potential advantage of circle-tracing as a measure of subtle cognitive decline is its apparent independence from general cognitive ability; in Insight 46, childhood cognitive ability has consistently predicted performance on diverse cognitive measures across the life-course (Lu *et al.*, 2019; Richards *et al.*, 2019), and hence its lack of association with circle-tracing performance is notable. Finally, this type of task has applicability to daily functioning, as visuomotor integration is intrinsic to many everyday activities—for example, subtle visuomotor difficulties may be relevant to declines in driving abilities in older adults with preclinical Alzheimer’s disease (Roe *et al.*, 2019).

Our finding that white matter disease was associated with poorer visuomotor integration—independently of amyloid—is consistent with the previous evidence relating lower white matter integrity to poorer visuomotor integration (Hawkins *et al.*, 2015). While participants were at an age where accelerated brain atrophy is still rare, we also found associations between brain volume and three diverse speed measures: circle-tracing speed, subtraction rate and Digit-Symbol Substitution [the latter reported by Lu *et al.* (2019)], consistent with the previous studies including in younger adults (Magistro *et al.*, 2015; Takeuchi *et al.*, 2017).

Interpretation of our finding of slower subtraction in both preclinical groups is limited by the fact that we did not measure subtraction in isolation in Insight 46, and hence cannot quantify the extent to which this result reflects poorer subtraction ability versus ‘dual-task cost’, which has previously been proposed as a sensitive marker of preclinical Alzheimer’s disease (MacPherson *et al.*, 2012). We hypothesize that both had an impact, given that FAD mutation carriers under-performed on an arithmetic task with a speed element, and showed dual-task costs on circle-tracing speed (Supplementary Material). This will be addressed in the next phase of Insight 46 with direct evaluation of single-task circle tracing and subtraction.

Strengths and limitations relating to the Insight 46 cohort have been discussed previously, key strengths being its population-based nature, very narrow age range and large sample size, and limitations being that the sample is entirely white and biased towards those with socioeconomic and health advantages (James *et al.*, 2018; Lu *et al.*, 2019). The lack of measures of tau pathology (which will be addressed in longitudinal data collection) limits conclusions about the pathological basis for visuomotor changes. The relatively small sample size of the FAD sample, due to the rarity of FAD mutations, limited statistical power to detect differences between mutation carriers and non-carriers, and limits comparisons between the FAD and Insight 46 samples.

## Conclusion

In summary, this study provides novel evidence of common visuomotor integration deficits in familial and sporadic preclinical Alzheimer’s disease, several years before expected symptom onset. Visuomotor integration tasks may hold promise as sensitive measures of disease-related cognitive decline.

## Supplementary Material

Supplementary material is available at *Brain Communications* online.

## Supplementary Material

fcab003_Supplementary_Data

## Abbreviations

FAD =: familial Alzheimer’s disease
MRC =: Medical Research Council
NSHD =: National Survey of Health and Development
SUVR =: standardized uptake value ratio
WMHV =: white matter hyperintensity volume

## References

[fcab003-B1] Bartoli E , Caso F, Magnani G, Baud-Bovy G Low-cost robotic assessment of visuo-motor deficits in Alzheimer’s disease. IEEE Trans Neural Syst Rehabil Eng 2017; 25: 852–60. 28574362 10.1109/TNSRE.2017.2708715

[fcab003-B2] Brookmeyer R , Abdalla N Estimation of lifetime risks of Alzheimer’s disease dementia using biomarkers for preclinical disease. Alzheimer’s Dement 2018; 14: 981–8. 29802030 10.1016/j.jalz.2018.03.005 PMC6097953

[fcab003-B3] Day GS , Musiek ES, Roe CM, Norton J, Goate AM, Cruchaga C, et al Phenotypic similarities between late-onset Autosomal dominant and sporadic Alzheimer disease a single-family case-control study. JAMA Neurol 2016; 73: 1125–32. 27454811 10.1001/jamaneurol.2016.1236 PMC5025942

[fcab003-B4] Gorbet DJ , Sergio LE Preliminary sex differences in human cortical BOLD fMRI activity during the preparation of increasingly complex visually guided movements. Eur J Neurosci 2007; 25: 1228–39. 17331218 10.1111/j.1460-9568.2007.05358.x

[fcab003-B5] Hawkins KM , Goyal AI, Sergio LE Diffusion tensor imaging correlates of cognitive-motor decline in normal aging and increased Alzheimer’s disease risk. J Alzheimer Dis 2015; 44: 867–78. 10.3233/JAD-142079 25374102

[fcab003-B6] Hawkins KM , Sergio LE Adults at increased Alzheimer’s disease risk display cognitive-motor integration impairment associated with changes in resting-state functional connectivity: a preliminary study. J Alzheimer Dis 2016; 53: 1161–72. 10.3233/JAD-151137 27340846

[fcab003-B7] Hawkins KM , Sergio LE Visuomotor impairments in older adults at increased Alzheimer’s disease risk. J Alzheimer Dis 2014; 42: 607–21. 10.3233/JAD-140051 24919768

[fcab003-B8] James S-N , Lane CA, Parker TD, Lu K, Collins JD, Murray-Smith H, et al Using a birth cohort to study brain health and preclinical dementia: recruitment and participation rates in Insight 46. BMC Res Notes 2018; 11: 885. 30545411 10.1186/s13104-018-3995-0 PMC6293512

[fcab003-B9] Joshi A , Ringman JM, Lee AS, Juarez KO, Mendez MF Comparison of clinical characteristics between familial and non-familial early onset Alzheimer’s disease. J Neurol 2012; 259: 2182–8. 22460587 10.1007/s00415-012-6481-y PMC3442121

[fcab003-B10] Lane CA , Barnes J, Nicholas JM, Sudre CH, Cash DM, Parker TD, et al Associations between blood pressure across adulthood and late-life brain structure and pathology in the neuroscience substudy of the 1946 British birth cohort (Insight 46): an epidemiological study. Lancet Neurol 2019; 18: 942–52. 31444142 10.1016/S1474-4422(19)30228-5 PMC6744368

[fcab003-B11] Lane CA , Parker TD, Cash DM, Macpherson K, Donnachie E, Murray-Smith H, et al Study protocol: Insight 46—a neuroscience sub-study of the MRC National Survey of Health and Development. BMC Neurol 2017; 17: 75. 28420323 10.1186/s12883-017-0846-x PMC5395844

[fcab003-B12] Leung KK , Barnes J, Modat M, Ridgway GR, Bartlett JW, Fox NC, et al Brain MAPS: an automated, accurate and robust brain extraction technique using a template library. NeuroImage 2011; 55: 1091–108. 21195780 10.1016/j.neuroimage.2010.12.067 PMC3554789

[fcab003-B13] Liu C-C , Kanekiyo T, Xu H, Bu G Apolipoprotein E and Alzheimer disease: risk, mechanisms and therapy. Nat Rev Neurol 2013; 9: 106–18. 23296339 10.1038/nrneurol.2012.263 PMC3726719

[fcab003-B14] Lu K , Nicholas JM, Collins JD, James S-N, Parker TD, Lane CA, et al Cognition at age 70: life course predictors and associations with brain pathologies. Neurology 2019; 93: e2144–56. 31666352 10.1212/WNL.0000000000008534 PMC6937487

[fcab003-B15] Lu K , Nicholas JM, James S-N, Parker TD, Lane CA, Keshavan A, et al Increased variability in reaction time is associated with amyloid beta pathology at age 70. Alzheimer’s Dement Diagnosis. Assess Dis Monit 2020; 12: e12076. 10.1002/dad2.12076 PMC7416668 32789161

[fcab003-B16] MacPherson SE , Parra MA, Moreno S, Lopera F, Della Sala S Dual task abilities as a possible preclinical marker of Alzheimer’s disease in carriers of the E280A presenilin-1 mutation. J Int Neuropsychol Soc 2012; 18: 234–41. 22133015 10.1017/S1355617711001561

[fcab003-B17] Magistro D , Takeuchi H, Nejad KK, Taki Y, Sekiguchi A, Nouchi R, et al The relationship between processing speed and regional white matter volume in healthy young people. PLoS One 2015; 10: e0136386. 26397946 10.1371/journal.pone.0136386 PMC4580478

[fcab003-B18] Malone IB , Leung KK, Clegg S, Barnes J, Whitwell JL, Ashburner J, et al Accurate automatic estimation of total intracranial volume: a nuisance variable with less nuisance. Neuroimage 2015; 104: 366–72. 25255942 10.1016/j.neuroimage.2014.09.034 PMC4265726

[fcab003-B19] McDade E , Wang G, Gordon BA, Hassenstab J, Benzinger TLS, Buckles V, for the Dominantly Inherited Alzheimer Network, et al Longitudinal cognitive and biomarker changes in dominantly inherited Alzheimer disease. Neurology 2018; 91: E1295–306. 30217935 10.1212/WNL.0000000000006277 PMC6177272

[fcab003-B20] Mormino EC , Papp KV, Rentz DM, Donohue MC, Amariglio R, Quiroz YT, et al Early and late change on the preclinical Alzheimer’s cognitive composite in clinically normal older individuals with elevated β-amyloid. Alzheimer Dement 2017; 13: 1004–12. 10.1016/j.jalz.2017.01.018 PMC5573651 28253478

[fcab003-B21] Nagengast AJ , Braun DA, Wolpert DM Risk sensitivity in a motor task with speed-accuracy trade-off. J Neurophysiol 2011; 105: 2668–74. 21430284 10.1152/jn.00804.2010 PMC3118741

[fcab003-B22] O’Connor A , Karikari TK, Poole T, Ashton NJ, Lantero Rodriguez J, Khatun A, et al Plasma phospho-tau181 in presymptomatic and symptomatic familial Alzheimer’s disease: a longitudinal cohort study. Mol Psychiatry 2020; 1–10. 10.1038/s41380-020-0838-x PMC7612227 32665603

[fcab003-B23] Peternel L , Sigaud O, Babič J Unifying speed-accuracy trade-off and cost-benefit trade-off in human reaching movements. Front Hum Neurosci 2017; 11: 615. 29379424 10.3389/fnhum.2017.00615 PMC5770750

[fcab003-B24] Pilotto A , Padovani A, Borroni B Clinical, biological, and imaging features of monogenic Alzheimer’s disease. BioMed Res Int 2013; 2013: 1–9. 10.1155/2013/689591 PMC3860086 24377094

[fcab003-B25] Richards M , James S-N, Sizer A, Sharma N, Rawle M, Davis DHJ, et al Identifying the lifetime cognitive and socioeconomic antecedents of cognitive state: seven decades of follow-up in a British birth cohort study. BMJ Open 2019; 9: e024404. 10.1136/bmjopen-2018-024404 PMC6502022 31023749

[fcab003-B26] Roe CM , Stout SH, Rajasekar G, Ances BM, Jones JM, Head D, et al A 2.5-year longitudinal assessment of naturalistic driving in preclinical Alzheimer’s disease. J Alzheimer Dis 2019; 1–9. 10.3233/JAD-181242 PMC6488385 30958365

[fcab003-B27] Rogojin A , Gorbet DJ, Hawkins KM, Sergio LE Cognitive-motor integration performance is affected by sex, APOE status, and family history of dementia. J Alzheimer Dis 2019; 71: 685–701. 10.3233/JAD-190403 31424400

[fcab003-B28] Ryan NS , Nicholas JM, Weston PSJ, Liang Y, Lashley T, Guerreiro R, et al Clinical phenotype and genetic associations in autosomal dominant familial Alzheimer’s disease: a case series. Lancet Neurol 2016; 15: 1326–35. 27777022 10.1016/S1474-4422(16)30193-4

[fcab003-B29] Say MJ , Jones R, Scahill RI, Dumas EM, Coleman A, Santos RCD, et al Visuomotor integration deficits precede clinical onset in Huntington’s disease. Neuropsychologia 2011; 49: 264–70. 21094653 10.1016/j.neuropsychologia.2010.11.016

[fcab003-B30] Scahill RI , Ridgway GR, Bartlett JW, Barnes J, Ryan NS, Mead S, et al Genetic influences on atrophy patterns in familial Alzheimer’s disease: a comparison of APP and PSEN1 mutations. J Alzheimer Dis 2013; 35: 199–212. 10.3233/JAD-121255 PMC4982537 23380992

[fcab003-B31] Sudre CH , Cardoso MJ, Bouvy WH, Biessels GJ, Barnes J, Ourselin S Bayesian model selection for pathological neuroimaging data applied to white matter lesion segmentation. IEEE Trans Med Imaging 2015; 34: 2079–102. 25850086 10.1109/TMI.2015.2419072

[fcab003-B32] Takeuchi H , Taki Y, Nouchi R, Yokoyama R, Kotozaki Y, Nakagawa S, et al Global associations between regional gray matter volume and diverse complex cognitive functions: evidence from a large sample study. Sci Rep 2017; 7: 10014. 28855703 10.1038/s41598-017-10104-8 PMC5577279

[fcab003-B33] Tang M , Ryman DC, McDade E, Jasielec MS, Buckles VD, Cairns NJ, et al Neurological manifestations of autosomal dominant familial Alzheimer’s disease: a comparison of the published literature with the Dominantly Inherited Alzheimer Network observational study (DIAN-OBS). Lancet Neurol 2016; 15: 1317–25. 27777020 10.1016/S1474-4422(16)30229-0 PMC5116769

[fcab003-B34] Tippett WJ , Krajewski A, Sergio LE Visuomotor Integration Is Compromised in Alzheimer’s disease patients reaching for remembered targets. Eur Neurol 2007; 58: 1–11. 17483579 10.1159/000102160

[fcab003-B35] Tippett WJ , Sergio LE Visuomotor integration is impaired in early stage Alzheimer’s disease. Brain Res 2006; 1102: 92–102. 16797495 10.1016/j.brainres.2006.04.049

[fcab003-B36] Vaportzis E , Georgiou-Karistianis N, Churchyard A, Stout JC Effects of task difficulty during dual-task circle tracing in Huntington’s disease. J Neurol 2015; 262: 268–76. 25371018 10.1007/s00415-014-7563-9

[fcab003-B37] Vaportzis E , Georgiou-Karistianis N, Stout JC Age and task difficulty differences in dual tasking using circle tracing and serial subtraction tasks. Aging Clin Exp Res 2014; 26: 201–11. 24136448 10.1007/s40520-013-0151-5

[fcab003-B38] Villemagne VL , Burnham S, Bourgeat P, Brown B, Ellis KA, Salvado O, et al Amyloid β deposition, neurodegeneration, and cognitive decline in sporadic Alzheimer’s disease: a prospective cohort study. Lancet Neurol 2013; 12: 357–67. 23477989 10.1016/S1474-4422(13)70044-9

[fcab003-B39] Vöglein J , Paumier K, Jucker M, Preische O, McDade E, Hassenstab J, Dominantly Inherited Alzheimer Network, et al Clinical, pathophysiological and genetic features of motor symptoms in autosomal dominant Alzheimer’s disease. Brain 2019; 142: 1429–40. 30897203 10.1093/brain/awz050 PMC6735903

