# Supplementary material for: Visuomotor integration deficits are common to familial and sporadic preclinical Alzheimer’s disease
Source: Brain Commun. 2021 Jan 25;3(1):fcab003. doi: 10.1093/braincomms/fcab003 (PMC7882207; doi:10.1093/braincomms/fcab003)
Supplement: fcab003_Supplementary_Data [file fcab003_supplementary_data.pdf]

Supplementary Material

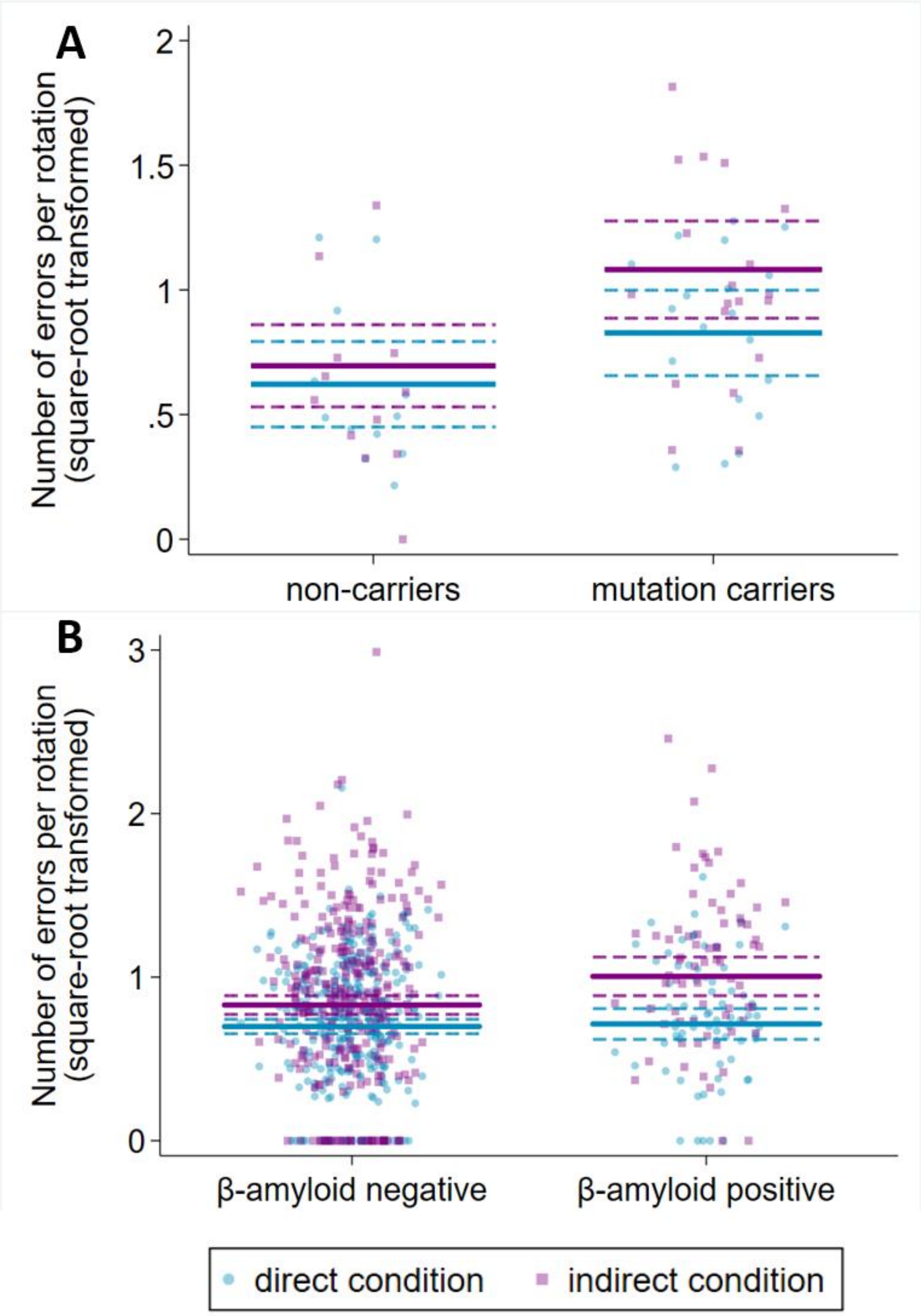

Supplementary Figure 1. Circle-tracing errors in the conditions of direct and indirect visual feedback for (A) Familial Alzheimer's disease mutation carriers (n = 19) vs. non-carriers (n = 12), (B) amyloid-negative (n = 318) vs. amyloid-positive (n = 72) Insight 46 participants.

The increase in errors in the indirect condition (compared to the direct condition) was greater in mutation carriers than non-carriers, and was greater in amyloid-positive than amyloid-negative participants. Solid lines show marginal means and dashed lines show 95% confidence intervals for the direct (in blue) and indirect (in purple) visual feedback conditions. Note that these means and confidence intervals are as shown in Figure 2 in the main paper, but individual-level data are added here for interest: markers show each participant's mean error rate for direct trials and indirect trials.

Dual-task cost in FAD

As the FAD participants completed both dual- and single-task circle-tracing trials (with and without concurrent serial subtraction), their tracing performance can be compared between the two. Results presented in the main manuscript show that tracing was slower in the dual-task, but more accurate (Table 1, Table 2). The improvement in accuracy can be explained by speed-accuracy trade-offs: as the two tasks competed for cognitive resources, participants were obliged to slow down, limiting the likelihood of adopting a hasty (i.e. fast but inaccurate) tracing style.

The reduction in speed can be quantified for each individual as a 'dual-task cost', which is a measure of the extent to which tracing speed was compromised by having to allocate cognitive resources to the subtraction task. Dual-task cost was calculated from the mean number of rotations completed in single- and dual-task trials (combined across the direct and indirect conditions) as follows: *(single – dual) / single*. A linear regression model was fitted where the outcome was dual-task cost and predictors were age, sex, education and mutation status (carrier *vs.* non-carrier). As before, the model was refitted in mutation carriers alone with an additional predictor of years to expected onset.

Dual-task cost for mutation carriers and non-carriers was as follows (mean (SD)): carriers = 0.14 (0.24); non-carriers = 0.15 (0.20). This means that tracing speed in the dual task was ~15% slower than in the single task. There was no group difference between mutation carriers and non-carriers (regression coefficient 0.03 [95% CIs -0.16, 0.21], $p = 0.78$). Among mutation carriers, there was a trend towards an association between closer proximity to expected onset and greater dual-task cost, but this was not statistically significant (regression coefficient = 0.028 per year [95% CIs -0.004, 0.061], $p = 0.083$).

Comparison of performance in the FAD and Insight 46 samples

Table 1 in the main manuscript presents descriptive statistics for the four task outcomes in the FAD and Insight 46 cohorts. Wilcoxon rank-sum tests were used to test for statistically-significant differences between the two cohorts in the dual-task condition (as Insight 46 participants were not administered single-task circle-tracing).

The only outcome with a statistically significant difference was circle-tracing speed (number of rotations), where FAD participants performed significantly faster ($z = 4.2$, $p < 0.0001$). This difference is likely to be age-related (FAD participants were 31 years younger than Insight 46 participants on average), consistent with a previous circle-tracing study that compared younger and older adults

(Vaportzis *et al.*, 2014), but could also partly be due to the fact that FAD participants may have been advantaged by practice effects, as they completed six single-task circle-tracing trials prior to this.

Performance of FAD participants on standard neuropsychological tests

Wilcoxon rank-sum tests were used to compare the performance of mutation carriers and non-carriers on a comprehensive battery of standard neuropsychological tests covering memory, language, executive function, visuospatial function, and general cognitive ability. Overall, they performed similarly; the only measures where mutation carriers showed evidence of poorer performance were Graded Difficulty Arithmetic, Block Design, and Spatial Span (**Supplementary Table 1**). These are measures that have some commonalities with circle-tracing and serial subtraction in terms of the tasks demands:

- Block Design requires participants to reproduce a pattern using blocks that have various colour patterns on different sides; as such, it requires spatial visualization and visuomotor co-ordination, somewhat similar to the spatial transformation demands of circle-tracing with indirect visual feedback.
- Spatial span is measured with a block-tapping task that requires participants to observe the researcher tapping a sequence of blocks (using an array of 9 identical spatially-separated blocks) and then to repeat the sequence either forwards (in the same order) or backwards (in reverse order). This is primarily a measure of visuospatial working memory, but the backwards condition is somewhat analogous to circle-tracing with indirect visual feedback in its requirement for spatial transformation.
- Graded Difficulty Arithmetic (addition and subtraction) has a 10-second cut-off for each response, so the outcome (number of correct responses) includes an element of speed as well as accuracy.

The effect sizes of the group differences on these tests may be compared by expressing the difference between the means of mutation carriers and non-carriers as a proportion of the standard deviation for the whole sample. These effects sizes are all similar in magnitude: Graded Difficulty Arithmetic 0.90 SD; Spatial Span forwards 0.87 SD; Spatial Span backwards 0.82 SD; Block Design 0.90 SD. Comparing this with the difference in serial subtraction and circle-tracing accuracy reported in the main manuscript, this is similar to the effect size for serial subtraction rate (0.87 SD), but the effect size for circle-tracing accuracy is somewhat higher (1.09 SD). This suggests that the circle-tracing task is at least as sensitive as these other tasks to the cognitive consequences of preclinical Alzheimer's disease pathology in FAD mutation carriers.

Supplementary Table 1. Performance of FAD participants on standard neuropsychological tests: mean (SD)

	**mutation carriers (n = 19)**	**non-carriers (n = 12)**	***p-value***
Camden paired associate learning /24	17.7 (4.4) [a]	19.6 (4.4) [a]	0.19
Digit Span Forwards – total correct /12	9.1 (2.2)	10.1 (1.7)	0.24
Digit Span Forwards – max length /8	7.1 (1.0)	7.3 (0.9)	0.79
Digit Span Backwards – total correct /12	7.7 (2.2)	8.7 (2.3)	0.35
Digit Span Backwards – max length /7	5.3 (1.3)	5.8 (1.2)	0.38
Digit Symbol Substitution Test /93	59.6 (14.4) [c]	65.3 (15.9)	0.46
Graded Difficulty Arithmetic /24	12.5 (5.6)	17.8 (5.0) [a]	**0.02***
National Adult Reading Test /50	26.3 (8.5) [a]	30.3 (4.6) [a]	0.42
Recognition Memory Test words /50	49.3 (0.9)	48.5 (2.3) [a]	0.32
Recognition Memory Test faces /50	44.5 (4.1)	42.6 (5.4) [a]	0.37
Spatial Span forwards – total correct /16	8.1 (1.8) [b]	10.1 (2.6) [a]	**0.03***
Spatial Span forwards – max length /9	5.8 (1.2) [b]	6.2 (1.5) [a]	0.46
Spatial Span backwards – total correct /16	8.3 (2.1) [b]	10.0 (1.7) [a]	**0.02***
Spatial Span backwards – max length /9	5.8 (1.2) [b]	6.6 (1.0) [a]	**0.03***
Trails A (time to complete - seconds)	24.2 (8.7) [b]	21.4 (6.3)	0.22
Trails B (time to complete - seconds)	57.4 (27.6) [b]	54.9 (24.9)	0.81
Verbal category fluency – animals (responses in one minute)	24.1 (7.0) [b]	26.6 (6.1)	0.33
Verbal phonemic fluency – letter F (responses in one minute)	16.9 (5.1) [b]	18.3 (6.2)	0.64
Visual Object & Space Perception – Object Decision /20	18.2 (1.4) [a]	18.4 (1.4) [a]	0.89
WASI Vocabulary /80	58.0 (10.6)	63.9 (5.9) [a]	0.12
WASI Similarities /48	35.8 (5.0)	37.2 (6.6) [a]	0.42
WASI Block Design /71	44.8 (11.6)	56.9 (13.4) [a]	**0.01***
WASI Matrix Reasoning /35	26.1 (4.9)	28.3 (3.4) [a]	0.21
WASI Verbal IQ	100 (14.0)	108 (14.2) [a]	0.12
WASI Performance IQ	107 (12.4)	118 (13.0) [a]	**0.03***

***significant difference between mutation carriers and non-carriers ($p < 0.05$), based on Wilcoxon rank-sum test. [a] One participant from this group was missing data for this test. [b] Two participants from this group were missing data for this test. [c] Three participants from this group were missing data for this test. WASI = Wechsler Abbreviated Scale of Intelligence*
